# Farm conditions shape microbial communities and their association with methane intensity in dairy cattle: insights from the rumen microbiome at the community level

**DOI:** 10.3389/frmbi.2025.1540197

**Published:** 2025-04-30

**Authors:** Simon Roques, Lisanne Koning, Alex Bossers, Sanne van Gastelen, Dirkjan Schokker, Edoardo Zaccaria, Léon Šebek, Soumya K. Kar

**Affiliations:** ^1^ Department of Animal Nutrition, Wageningen Livestock Research, Wageningen, Netherlands; ^2^ Department of Epidemiology, Bioinformatics, Animal Models & Vaccine Development, Wageningen Bioveterinary Research, Lelystad, Netherlands; ^3^ Institute for Risk Assessment Sciences (IRAS), Utrecht University, Utrecht, Netherlands

**Keywords:** dairy cattle, emission, GreenFeed system, living lab, methane, microbiome, rumen

## Abstract

Rumen microbial communities are known to drive methane (CH_4_) production, but their dynamics in variable “real-world” farming environments are less understood. This research aims to identify specific microbial taxa linked to CH_4_ emission in commercial dairy farms by employing 16S rRNA gene sequencing, thereby providing a more ecologically relevant understanding of CH_4_ production in real-world settings.Rumen fluid samples were collected from 212 cows across seventeen Dutch dairy farms. Methane production was measured from these dairy cows using the GreenFeed system and expressed as CH_4_ intensity (g fat- and protein-corrected milk yield^−1^). Rumen microbiota was analyzed using 16S rRNA gene amplicon sequencing. Analysis was performed to assess association between microbial taxa and CH_4_ intensity, using data from individual cattle across the dairy farm. We observed that diet and lactation stage influenced CH_4_ intensity, consistent with previous studies. Results showed higher CH_4_ intensity in cows during late lactation, and feeding type, particularly fresh grass intake, strongly influenced rumen microbiota. However, after classifying low and high CH_4_ emitting cows, only limited differences in microbiota composition could be measured. Few taxa, like *Lachnospiraceae*, were common across both groups, while *Ruminoccocaceae* and *Rikenellaceae* were more abundant in low emitters, and *Oscillospiraceae* in high emitters. *Methanobrevibacter* differentiated CH_4_ emission groups, but archaeal methanogen abundance may not accurately reflect CH_4_ variation due to methodological limitations, including reliance on relative abundance, limited taxonomic resolution, and primer bias. Using a bacterial-biased 16S rRNA approach, we observed a limited number of consistent bacterial taxa associated with CH_4_ intensity highlights the challenges of studying dairy farms under practical conditions, where variability in diet, genetics, and management practices complicates the identification of specific rumen microbes associated with CH_4_ emission. Even with control over key variables, the inherent variability of on-farm conditions impeded the detection of stable microbial patterns. In conclusion, our study clearly indicates that understanding CH_4_ emissions from dairy cattle in real-world settings fundamentally requires a broader ecological perspective where rumen microbes are recognized as key determinants influencing microbiota signals within multi-factorial farm settings.

## 1 Introduction

The rumen microbiome, a complex consortium of microorganisms, is integral to CH_4_ production. Enteric CH_4_ is produced during the fermentation of feed carbohydrates by microbiota in the rumen. Archaea play a major role in the production of enteric CH_4_ as they are the only microorganism capable of producing CH_4_ in the rumen (Morgavi et al., 2010). Within archaea, CH_4_ is produced through the reduction of CO_2_ and methanol, as well as a few minor substrates, with hydrogen (H_2_) serving as the primary electron donor (Kurth et al., 2020; Tapio et al., 2017). However, CH_4_ production also depends on other microbes, as the necessary substrates are produced by other microbes within the rumen (Mizrahi and Jami, 2018). Bacteria, for instance, produce H_2_ (e.g. *Ruminococcaceae*, *Eubacterium* spp and numerous Firmicutes) (Tapio et al., 2017) or even methanol during pectin fermentation (some *Lachnospiraceae*) (Li, 2021). On the opposite, some microbes consume H_2_ (e.g., Fibrobacter succinogenes) and compete with methanogenic archaea for H_2_; but to date, the uptake of H_2_ by methanogen is still thermodynamically more favorable (Ungerfeld, 2020).

Overall, the association between the rumen microbiome and CH_4_ emission is a key area of research to identify the microorganisms associated with enteric CH_4_ emission. The rumen microbiota is a dynamic community shaped by ecological interactions within the rumen and influenced by a range of host-related and external factors. For instance, the rumen microbiota and its association with CH_4_ production is shaped by factors like diet (Huws et al., 2018), genotype (Difford et al., 2018) and lactation stage of the animals (Bainbridge et al., 2016). Traditionally, controlled experimental setups are used to minimize the variation induced by factors of less interests (e.g., cows fed the same diet to identify microbial biomarkers related to CH_4_ emission in specific breeds; Ramayo-Caldas et al., 2020). However, these controlled environments do not fully reflect the complexity of “real-world-” or “commercial-” or “practical-” farming conditions.

In real-world conditions, ruminants are exposed to highly variable environments. For instance, diets can vary significantly between farms, even within the same region, with differences in quality and the proportions of roughages (such as fresh grass, grass silage, and corn silage), concentrates, and by-products. Some farms allow their cows to graze, with grasslands varying not only between farms but also throughout the year due to differences in botanical composition (Totty et al., 2013), weather conditions, and fertilization practices (Rinne et al., 1997). Furthermore, animal characteristics on commercial farms are not standardized, unlike in controlled experimental settings where these factors are carefully managed to minimize variability.

Cows on commercial (practical) farms display a much broader range of variation in genetics, parity, and lactation stage, which could potentially lead to different outcomes than those observed in controlled experiments. Large-scale studies analyzing rumen microbiota across different farms rarely focus on the association with CH_4_ emission (Xue et al., 2018). However, studies that do examine both microbiota and CH_4_ emission, provide valuable insights into the relation between rumen microbes and CH_4_ emission under real-world-farming conditions (Difford et al., 2018). The lessons and insights gained from such studies are increasingly important for the future, especially with the emergence and adoption of research concepts like the “living labs” approach for dairy cattle (Karlsson et al., 2024; Pasinato et al., 2023). Therefore, this study aimed to identify specific microbial taxa associated to CH_4_ emission in dairy cows raised under real-world farming conditions.

## 2 Materials and methods

### 2.1 Animals, housing, and diet

The study was conducted from September 2018 to March 2020 on 17 dairy farms in the Netherlands. In this time frame, farms were visited one to three times (**Supplementary Table 1**), each time for a period of at least four weeks; a minimum of two weeks of adaptation followed by two weeks of measurement period. The farms were selected from the project “*Koeien & Kansen*”, a multi-annual research and demonstration project in the Netherlands that aims to be representative of the Dutch dairy sector. The study was conducted in accordance with the Dutch Animal Experiments Act in compliance with European Union Directive 2010/63 and approved by the Central Committee for Animal Experiments (The Hague, The Netherlands, 2016.D-0066.001).

In total 212 dairy cows were sampled in this study, of which 15 cows were measured multiple times over the study period with a maximum of three times. Because there was considerable time between each measurement period, including changes in diet, lactation stage and parity, observations of the same cows were considered to be independent. All cows were housed in free-stall barns with deep-litter sand or rubber matrass with sawdust or in a (composted) bedded pack barn. All barns had open sides for natural ventilation and free access to clean drinking water. All cows were fed ad libitum and received a commercial dairy diet that differed per farm and season. The diets were formulated according to commercial practices complied in collaboration with a dairy feed advisor. Practices and diets per farm were maintained unchanged during the adaptation period and measurement period as the objective was to have a representative overview of Dutch farming on CH_4_ emission and rumen microbiota. On average (mean ± SD) the diets consisted of 32 ± 13.6% grass silage, 25 ± 11.8% maize silage, 12 ± 13.8% fresh grass, 25 ± 7.9% concentrates, and 6 ± 10.7% by-products, on a DM basis. **Supplementary Table 1** presents the diet composition provided on each farm during each measurement period. Fifteen farms applied grazing during part of the year (spring, summer and/or autumn) and thus also during some of the measurement periods. Grazing time was not controlled and differed per farm, ranging from a few hours a day to unrestricted grazing with free choice to stay outside the barn. Farmers were advised to keep the grazing management as homogenous as possible during the measurement period.

### 2.2 Milk measurements

Depending on farm, cows were milked either twice daily in a milking parlor or two to three times daily by a milking robot. Milk composition was determined at the individual cow level using milk samples collected on a single day in the second measurement week. Milk samples were analyzed by Fourier transform mid-infrared spectrometry (MIRS) as part of the routine milk recording programs, for 15 farms performed by Qlip B.V. (Zutphen, the Netherlands) and for 2 farms by VVB Veluwe-Ijsselstreek (Nunspeet, the Netherlands). Milk yield and the percentage of protein and fat in the milk of a sample day were used to calculate fat- and protein-corrected milk (FPCM) according to the following equation (CVB, 2016).


$$\text{FPCM }\left(\text{kg}.{\text{d}}^{-1}\right)=\left(0.337+0.16\times \%\text{fat}+0.06\times \%\text{protein}\right)\times \text{milk yield }\left(\text{kg}.{\text{d}}^{-1}\right)$$

### 2.3 Methane measurements

Enteric CH_4_ production was measured non-invasively using the GreenFeed system (GF, C-lock Inc. Rapid City, SD, USA). The GF is an adapted feeding station that measures individual CH_4_ and CO_2_ production in grams per day during each visit, as described in detail by van Gastelen et al. (2022). For the present study, the average recovery of CO_2_ was 99.1% (for individual GF systems between 97.6 and 102.7%).

Methane production was measured over a 14-day period, with a minimum 7-day acclimation to the GF prior to the measurement period and visit times per cow ranging from 3 to 6 min. Cows received 2 to 6 kg of compound feed per day via the GF, depending on milk yield and lactation stage. Administration of the compound feed was divided into 4 to 6 feeding periods per day, with at least 3 to 4 h between each feeding period. Per feeding period, 0.5 to 1 kg of compound feed was administered in 12–25 drops of approximately 40 g of feed (ranging between 31 and 51 g per drop, depending on the type of compound feed used). There were 10–30 s between each drop (depending on the maximum number of drops) to ensure a minimum visit time of 3 min, but no longer than 6 min.

On each farm, one GF was installed in the barn. In case grazing was applied at farm level, an additional GF on a pasture trailer was used to ensure adequate CH_4_ measurements throughout the day. Due to the high diurnal variation of enteric CH_4_ production and using short-term breath measurements, multiple records are needed to provide a representative average. Therefore, based on what was found in the study of Manafiazar et al. (2017), CH_4_ measurements from the 14-day measurement period were averaged per cow and 32 cows with fewer than 20 valid records were excluded from the analysis. Methane intensity (g CH_4_.kg FPCM^−1^.d^−1^) was used as a metric for CH_4_ emission rather than CH_4_ yield (g CH_4_.kg DM^−1^.d^−1^). Methane production (g.d^−1^) is strongly related to total feed intake, which makes it necessary to take measures of feed intake into account when comparing CH_4_ emission. However, feed intake was not available on individual cow level, thus CH_4_ production was corrected for FPCM yield, as it is the closest available measure related to feed intake. Note that CH_4_ production was averaged over two weeks while FPCM yield resulted from a single day of milk samples.

### 2.4 Rumen sampling

Rumen fluid samples were collected in the second week of the measurement period (**Supplementary Table 1**) using the oral stomach tube (OST) (Muizelaar et al., 2020). Briefly, the OST consisted of a manual pump and a 190 cm long spiral probe with a perforated suction head at the end, inserted through the esophagus into the dorsal cranial part of the rumen. Rumen fluid was collected by the manual pump. The first 500 ml of the rumen fluid was discarded to minimize contamination with saliva. Subsequently, samples of 3 ml were collected and immediately frozen in dry ice. Samples were stored at –80°C until analysis.

### 2.5 Microbial DNA extraction and sequencing

Extraction of microbial DNA from rumen samples, library construction of hypervariable region V4 (from 16S rRNA gene) and subsequent sequencing on an Illumina HiSeq platform were performed at Genotypic Technology Pvt. Ltd. In Bangalore, India.

DNA from rumen fluid samples was purified using the Qiagen Dneasy Blood and tissue Kit (Qiagen, Hilden, Germany). Prior to processing the samples using this method, approximately 200μl of ruminal fluid was taken in sterile Tomy tubes containing 3–4 beads and homogenization was carried out at 4,000 rpm for 120 s. A volume of 200 μl (10mg ml^−1^) lysozyme (MilliporeSigma, St. Louis, USA) was added to the homogenates. The homogenate tube was invert mixed and incubated for 30 min at 37°C. A volume of 200μl AL buffer was added to the samples and vortexed briefly. The samples were subjected to Proteinase K treatment at 56°C for 2 hours followed by Rnase A treatment (MP Biomedicals, Solon, USA) at 65°C for 20 min. The lysate was mixed well with 100% ethanol and loaded onto Qiagen Dneasy blood and tissue column (Qiagen, Hilden, Germany). The samples were further processed following manufacturer’s instructions. Finally, DNA was eluted in 45μl 10mM Tris.Cl, pH 8.0 (Sigma-Aldrich, St. Louis, USA). The concentration and purity of genomic DNA was quantified using the Nanodrop Spectrophotometer (Thermo Scientific; 2000). The integrity of the DNA was assessed by agarose gel electrophoresis.

Sequencing libraries were prepared by a two-step polymerase chain reaction (PCR)-based workflow based on primers specific to V4 region of the 16S rRNA gene (Caporaso et al., 2011). In the first round of PCR, the V4 region of the 16S rRNA gene was amplified using the region-specific primers V4-515F (GTGCCAGCMGCCGCGGTA) and V4-806R (GACTACHVGGGTATCTAATCC) designed by Genotypic Technology Pvt. Ltd. In Bangalore, India. Using the KAPA HiFiHotStart PCR Kit (KAPA Biosystems Inc., Boston, MA USA) and a primer concentration of 5μM, 50ng of genomic DNA was amplified by following first round of PCR condition: 3 min at 95°C, 26 × (30 s at 95°C, 30 s at 64°C, and 30 s at 72°C), and 5 min at 72°C. The amplicons generated were analyzed on a 1.2% agarose gel. The second round of PCR was performed to index the amplicons generated in the first round of PCR. 1μl of the 1:2 diluted PCR amplicons generated in the first round were amplified by following second round of PCR condition: 3 min at 95°C, 10 × (30 s at 95°C, 30 s at 55°C, and 30 s at 72°C), and 5 min at 72°C to add Illumina barcoded adaptors for sequencing (Nextera XT v2 Index Kit, Illumina, USA). Amplicons (sequencing libraries) generated by the second round of PCR were analyzed on a 1.2% agarose gel. The libraries were normalized and pooled for high-throughput multiplex sequencing. Finally, these pools were quantified using the Qubit dsDNA HS assay and fluorometer (Thermo Fisher Scientific, MA, USA). The normalized sample was denatured and fed into the Illumina HiSeqXTen sequencer, where it was sequenced for 150*2 cycles to generate at least 0.7 million paired-end reads. Upon completion of the sequencing run, the data were demultiplexed using bcl2fastq v2.20 software (Illumina, San Diego, USA).

### 2.6 Sequences pre-processing

Reads were preprocessed using QIIME2 suite v2020.8 (Bolyen et al., 2019). Reads were first checked for low quality bases (Q30) with FAST QC v 0.11.9, then trimmed of primers with Cutadapt plug-in before being merged using FASTQ-join. The merged reads were denoised, dereplicated, and chimera sequences were removed using the DADA2 plugin (Callahan et al., 2016) with default parameters from the QIIME2 suite. The resulting Amplicon Sequence Variants (ASV) were classified based on SILVA v.138 database (Quast et al., 2013). To achieve the classification a Naive Bayesian classifier, pre-trained, was used with the ‘feature-classifier classify-sklearn’ command implemented in Qiime2 (Bokulich et al., 2018). The classifier was optimized for 515F/806R region at 99% similarity and can be found here: https://resources.qiime2.org/#naive-bayes-classifiers-2. Confidence level was set at 0.7% by default.

### 2.7 Balanced experimental design

**Figure 1:**
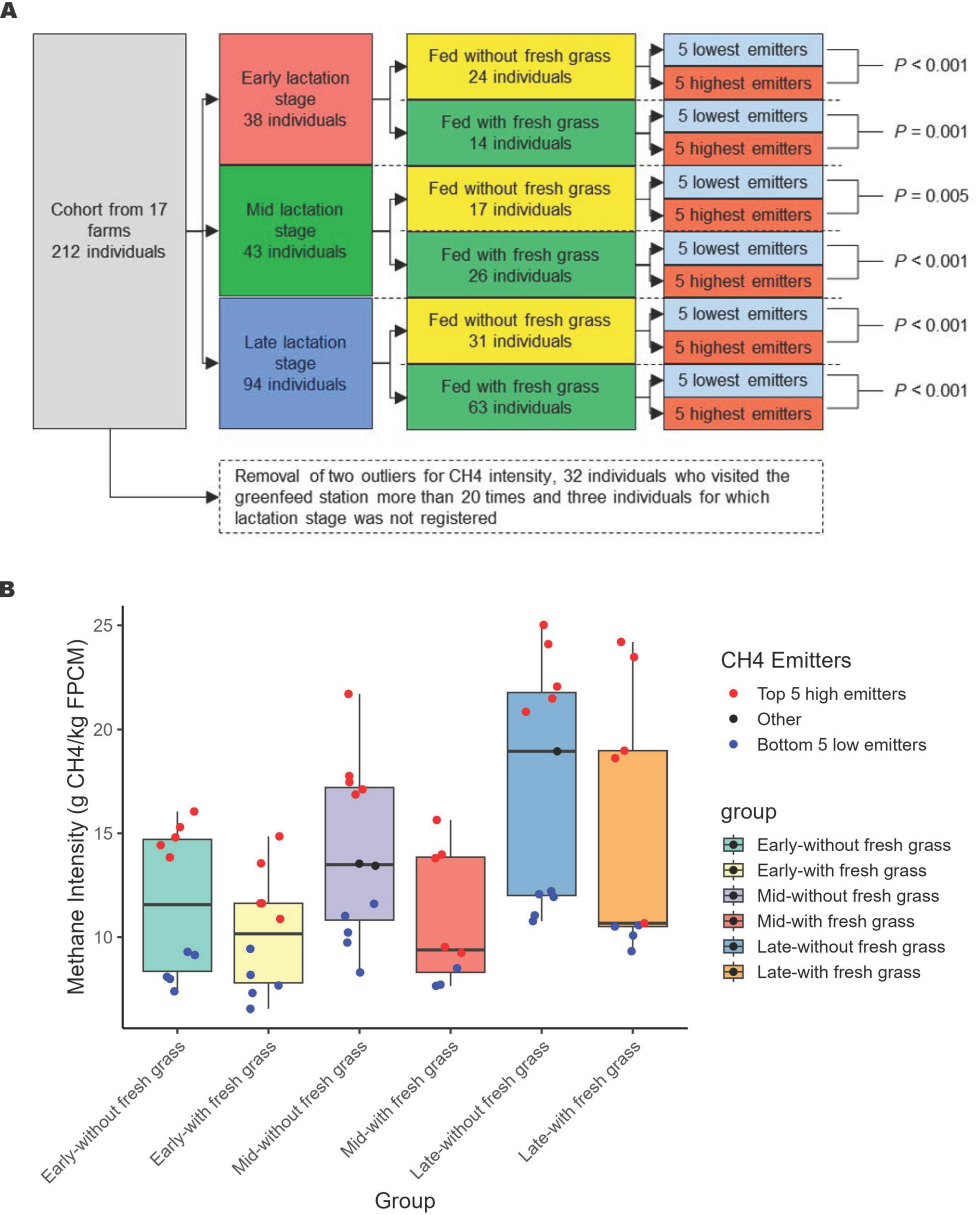
Selecting high and low methane (CH_4_) emitters from the cohort of 212 cows. **(A)** provides a schematic representation of the balanced experimental design. Cows were initially stratified by lactation stage and further grouped by diet (with or without fresh grass). From each of the six resulting subgroups, the five lowest and five highest methane emitters were selected, forming balanced “low” and “high” emitter groups of 30 cows each. The groups were balanced for lactation stage and diet. Statistical significance in CH_4_ intensity differences was evaluated using Welch’s t-test, with the p-value reported. **(B)** displays the mean CH_4_ intensity for each subgroup, categorized by lactation stage (early, middle, or late) and diet type (with or without fresh grass). The top five highest emitters (in red) and the bottom five lowest emitters (in blue) within each subgroup were selected to form the “high” and “low” emitting groups, respectively.

A balanced experimental design was established from this large cohort after data generation to balance the factors influencing CH_4_ intensity and rumen microbiota (**Figure 1A**). First, two dairy cows with outlier CH_4_ intensity (above third quartile added of three times the interquartile range) were removed from the dataset. Second, dairy cows were grouped into three lactation stages, namely early (0–93 days in milk (DIM)), mid (93–183 DIM) and late (>183 DIM), according to Bainbridge et al. (2016). Third, dairy cows within each lactation stage were split in two dietary categories, a group that had access to fresh grass during the measurement period and a group that had not access to fresh grass during the measurement period. This resulted in six groups. Fourth, the top fifth lowest and top fifth highest CH_4_ emitters within each of these six groups were selected (**Figure 1B**), after checking for statistical significance of the difference of their mean CH_4_ intensity by a Welch test (**Figure 1A**), and placed in two groups; a low and high CH_4_ emitting group (N=30 per group) that was balanced for lactation stage and diet type (i.e., with or without access to fresh grass). All the high and low emitters cows within this experimental design were either Holstein Friesian or Holsten Friesian cross breed.

### 2.8 Statistical analysis

All statistical analysis were performed in R v4.0.3 (R Core Team, 2020) using phyloseq (McMurdie and Holmes, 2013), vegan (Oksanen et al., 2012) and ropls (Thévenot et al., 2015) packages. Methane intensity was compared between low and high CH_4_ emitter groups by a Welch test. The low-high dataset previously described was filtered for low abundant and low prevalent taxa (more than five in at least a third of individuals) to limit the zero-inflation issue (from 90.1% 0 in the dataset prior filtering to 34.0% 0 after filtration). Relative abundance of ASVs were then transform by centered log-ratio (CLR) which allows to overcome differences in sequencing depth without wasting data as in rarefaction (McMurdie and Holmes, 2014; Gloor et al., 2017) and at the same time replace data into a Euclidean space (Quinn et al., 2019). Multivariate dimension reduction approaches, namely principal component analysis (PCA) and orthogonal partial least-square discriminant analysis (o-PLS-DA) were performed following the CLR normalization and a z-score scaling to adjust feature-wise homogeneity. Orthogonal signal correction was used to decorrelate variation that would be unrelated to the discriminant low and high emitter groups. Significance of the predictive performance (Q2) was assessed by random permutation of 10% of the samples in the discriminant groups repeated 200 times. Variables important for projection with a value above two were defined as associated with low or high emitters depending on their contribution on the predictive component. The relative abundance of taxa was used to plot the VIP rather than CLR transformed value for comprehension purpose.

## 3 Results

### 3.1 Methane emission and animal characteristics

**Figure 2:**
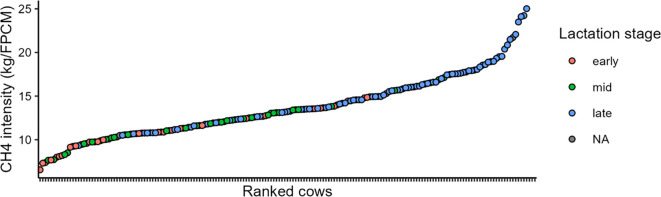
Methane emission of dairy cows from the cohort of 212 dairy cows representative of the Dutch cattle farming ranked from lowest to highest and depleted by two outliers and 32 cows that visited the greenfeed station less than 20 times.

Methane intensity from the broad cohort of 212 cows, depleted of two outliers and 32 cows that visited the GF less than 20 times, ranged from 6.55 to 25.02 g.kg FPCM^−1^.d^−1^ (**Figure 2**). The low CH_4_ emitters were predominantly cows in early or mid-lactation, whereas the high CH_4_ emitters were mostly cows in late lactation. To isolate individual effects on CH_4_ intensity from important confounding factors, we created a balanced experimental design that account for lactation stage (i.e., early, mid, and late) and diet type (i.e., with or without access to fresh grass) for two groups of low and high CH_4_ emitters. The average CH_4_ intensity in the low CH_4_ group (9.5 ± 1.57 g.kg FPCM^−1^.d^−1^) was significantly different from average CH_4_ intensity in the high CH_4_ group (16.8 ± 4.11 g.kg FPCM^−1^.d^−1^, P < 0.001). The group composition was not biased for specific farms (**Supplementary Figure 1**) and only one cow in each group was sampled twice.

### 3.2 Microbiota composition

The dataset from the balanced experimental design contained 23,578 ASV for 60 samples before filtering for low abundant and low prevalent ASV. After the filtering, 2,250 taxa remained. On average, 98.10 ± 2.10% of the ASV were identified as bacteria and 1.90 ± 2.10% were identified as archaea (**Supplementary Figure 2**). At the Phylum level, Firmicutes (56.6 ± 9.46%), Bacteroidota (31.90 ± 10.17%) and Proteobacteria (3.10 ± 3.55%) composed most of the taxa (**Supplementary Figure 3**).

**Figure 3:**
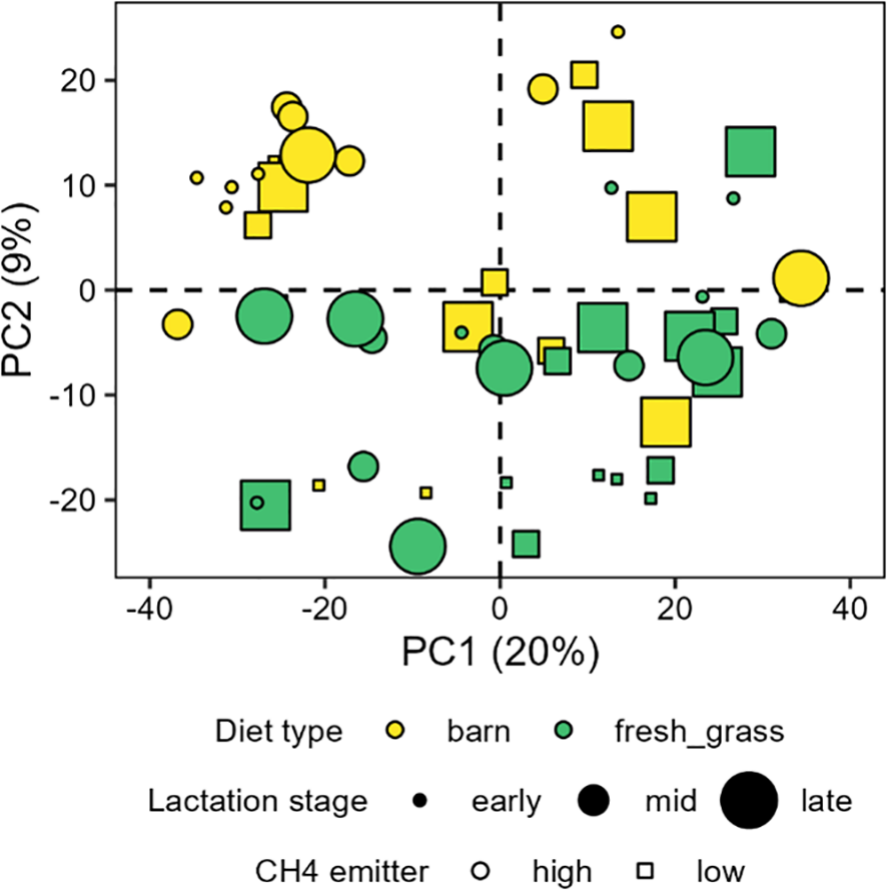
Principal component analysis scores plot of cow rumen fluid microbiota composition from the balanced design of 60 cows. Diet type, rather than lactation stage or CH_4_ emission category, structures the PCA.

The microbial composition at Phylum level across the 17 farms is presented in **Supplementary Figure 4**. In farm “I”, the relative abundance of the most abundant phylum, Firmicutes, varied considerably, ranging from a minimum of 47.12 ± 9.18% to a maximum of 71.04 ± 10.55%. A PCA was performed to have an overview of the main factors that influenced the composition of rumen microbiota of cows in the balanced experimental design (**Figure 3**). The first two principal components (PC) of the PCA explained 29% of the total variance. The most visible separation on the PCA, if any, was related to diet type, namely, the presence of fresh grass in the diet. No discrimination by the lactation stage or by the grouping of cows as low or high CH_4_ emitters was observed.

### 3.3 Microbiota composition differences between low and high emitters

**Figure 4:**
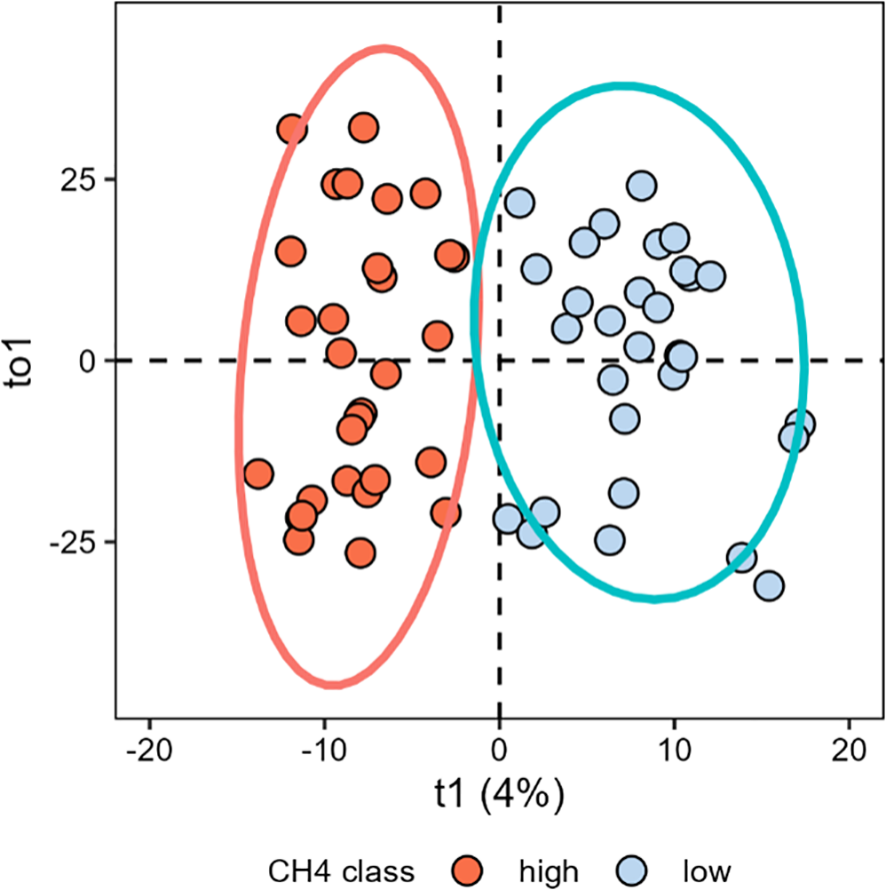
Orthogonal partial least square discriminant analysis (oPLS-DA) of rumen fluid from low and high emitters (N=30 per group) in the balanced experimental design from the cohort of dairy cattle. R2Y = 82%, Q2Y = 0.16, P = 0.01.

**Figure 5:**
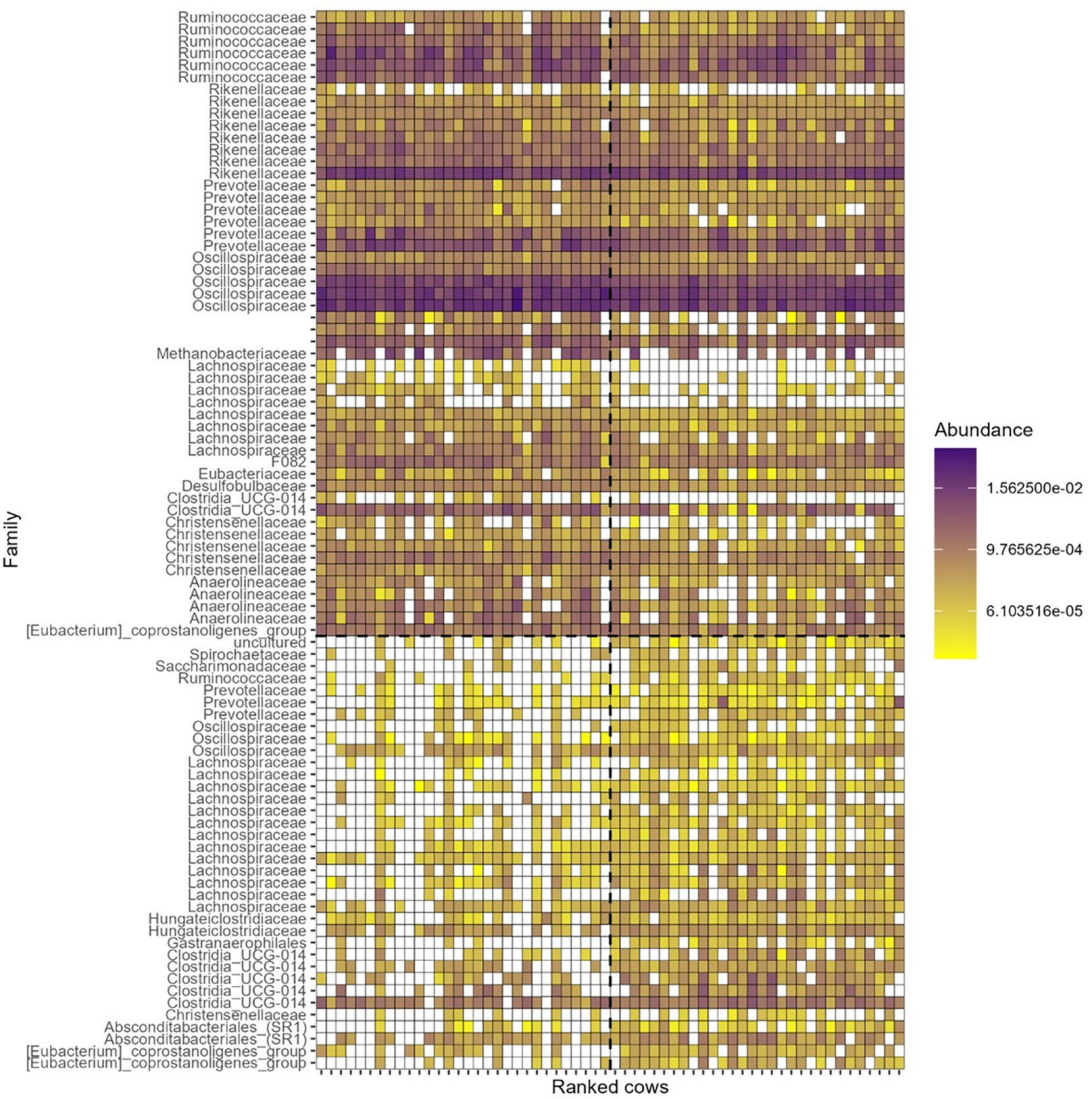
Heatmap of taxa with variable importance in projection (VIP > 2) identified in the oPLS-DA differentiating low and high methane emitters. Each cow is ranked from low (left side, 30 lowest) to high emission (right side, 30 highest). Each taxon is identified by its Family rank when possible (otherwise left unnoted) and ordered alphabetically within taxa more abundant in high emitters (bottom half) and more abundant in low emitters (top half). Abundance (0–1) within each cows’ rumen fluid is color coded.

A multivariate discriminant analysis, o-PLS-DA, was performed to reveal ASV associated with low and high CH_4_ emitter groups in the balanced experimental design (**Figure 4**). The predictive accuracy of the o-PLS-DA was 0.16 (Q2 = 0.16) and significant (P = 0.01). A total of 88 VIP (ASVs used in the model) were above the defined threshold of two, i.e., for VIP highly discriminant for the two groups (**Figure 5**). Collectively, these VIP represented 14.0 ± 4.9% of the total relative abundance across all the 60 samples of the balanced experimental design (**Supplementary Figure 5**). Thirty-six taxa were more abundant in the high CH_4_ emitter group than in the low emitter group, whereas 52 were more abundant in the low CH_4_ emitter group than in the high emitter group. The 36 ASV that were relatively more abundant in the high CH_4_ emitter group were identified in the family [Eubacterium]_coprostanoligenes, Absconditabacteriales_(SR1), *Christensenellaceae*, Clostridia_UCG−014, Gastranaerophilales, *Hungateiclostridiaceae*, *Lachnospiraceae*, *Oscillospiraceae*, *Prevotellaceae*, *Ruminococcaceae*, *Saccharimonadaceae*, *Spirochaetaceae* and one uncultured family from the Armatimonadota Phylum. The 52 ASV more abundant in low CH_4_ emitter group were identified in the family [Eubacterium]_coprostanoligenes, *Anaerolineaceae*, *Christensenellaceae*, Clostridia_UCG−014, *Desulfobulbaceae*, *Eubacteriaceae*, F082 from the Bacteroidales order, Lachnospiraceae, Methanobacteriaceae, unknown family from the Clostridia class, *Oscillospiraceae, Prevotellaceae*, *Rikenellaceae* and *Ruminococcaceae*.

## 4 Discussion

In this study, we investigated the association between microbial taxa and CH_4_ intensity, using data collected from individual cattle across seventeen Dutch dairy farms operating under practical farming conditions. As individual dry matter intake (DMI) measurements are often unavailable in such settings, CH_4_ intensity, defined as CH_4_ production per unit of fat- and protein-corrected milk (FPCM), was used as a proxy instead of CH_4_ yield, which is expressed as CH_4_ production per unit of DMI. During the analysis of the data in this study, the inherent complexities and variability in dairy cattle farm conditions and husbandry practices presented significant challenges in identifying consistent rumen microbial taxa associated with CH_4_ intensity as shown in the variation of the main Phyla (**Supplementary Figure 4**). We attempted to mitigated some of the variability by applying a balanced design post-data collection, carefully accounting for factors such as lactation stage and the availability of fresh grass, both of which are known to influence rumen microbiota, either directly or indirectly. Although the dynamic and diverse nature of our dataset complicates the identification of specific taxa with precision, our primary objective was to uncover consistent microbial patterns linked to the observed CH_4_ emission intensities. Therefore, while acknowledging the inherent complexity, our analysis focused on identifying robust microbial signatures that consistently associated with CH_4_ emission intensities, even if the precise taxonomic resolution remained challenging. As an outlook, future research could explore these broader ecological factors to gain a more comprehensive understanding of the microbial influences on CH_4_ production in diverse farm settings.

In a broader context, this study was part of a larger inventory study in which CH_4_ production was measured from 1,106 cows of 18 farms (Koning et al., 2020). The linear mixed model analysis conducted in the inventory study revealed that grazing, coupled with seasonal variations, accounted for approximately 3% of the variation in CH_4_ intensity. While the impact of fresh grass on CH_4_ emission was modest in the study of Koning et al. (2020), our study demonstrated a more pronounced effect of fresh grass on rumen microbiota while the effect of lactation stage was more ambiguous. Our findings highlight a complex, multifactorial relationship between grazing practices, rumen microbial dynamics, and CH_4_ emission under real-world farming conditions. Nonetheless, it is plausible that the binary classification of fresh grass availability may mask subtle yet significant dietary variations, which could intricately influence the structure and function of the rumen microbiota involved in CH_4_ production. These unaccounted dietary nuances may contribute to the observed variability in CH_4_ emission, underscoring the need for more granular assessments of feed composition and its microbial interactions.

Interestingly, our analysis revealed a notable similarity in the bacterial community composition within the rumen of both high and low CH_4_-emitting cows. One of the most predominant families identified was *Lachnospiraceae*, which was consistently present in both high and low emitters. Bacteria from the *Lachnospiraceae* have been associated with CH_4_ emission in ruminants in various studies (Ramayo-Caldas et al., 2020; Shabat et al., 2016; Kittelmann et al., 2014). They are known to produce H_2_ which is then used to reduce CO_2_ into CH_4_. The *Lachnospiraceae* family encompasses a range of species known for their diverse abilities to degrade plant polysaccharides, highly present in cow diets (Biddle et al., 2013; Sorbara et al., 2020). This metabolic versatility likely contributes to the widespread presence of *Lachnospiraceae* in the rumen of cows across different farms. The ability of this bacterial family to efficiently process a variety of plant-based diets enables its prevalence in bovine ruminal microbiomes, regardless of variations in the plant material composition in the feed across the 17 farms studied. Interestingly, Shabat et al. (2016) also reported that genes from the *Lachnospiraceae* family were enriched in cows both with a low or high feed efficiency, and subsequently a high or low CH_4_ production, respectively. In a recent study, Kaminsky et al. (2023) highlighted the flexibility of the NK3A20 isolate from the *Lachnospiraceae* family to grow from different substrates. They observed that this isolate exhibited varied H_2_ production depending on the substrate, with notably lower H_2_ production when metabolizing galacturonic acid. This could imply a reduced potential for CH_4_ production under certain dietary conditions. Based on these findings, it can be speculated that the presence of *Lachnospiraceae* in both the low and high CH_4_ emitting group may not solely be attributable to the variations in diet composition across farms. Instead, the inherent genetic and metabolic adaptability of this bacterial family, particularly in response to different substrates, could play a significant role in its prevalence and activity in varying CH_4_ production contexts. Overall, despite their divergent CH_4_ emission profiles in high and low CH_4_ emitters, the overall microbial structure remained largely conserved, suggesting that differences in CH_4_ emission may be driven by functional or metabolic shifts within the microbial community, rather than substantial changes in its taxonomic composition.

In our study, members of the *Ruminococcaceae* family were found to be more prevalent in low CH_4_-emitting cows, contrasting with prior research that frequently reported higher abundances of *Ruminococcaceae* in high CH_4_ emitters. This discrepancy suggests that the relationship between *Ruminococcaceae* and CH_4_ production may be context-dependent, potentially influenced by factors such as diet composition, host genetics, or environmental conditions, highlighting the complexity of microbial contributions to CH_4_ emission (Tapio et al., 2017; Ramayo-Caldas et al., 2020). *Ruminococcaceae* are known for their role in cellulose degradation and H_2_ production, their prevalence in low CH_4_ emitters in our study prompts further investigation. Furthermore, the absence of *Fibrobacteraceae*, particularly the absence of ASVs associated with *Fibrobacter succinogenes*, among the variables identified as important for projection (VIP), is noteworthy and warrants further investigation. *Fibrobacter* species are specialized in cellulose and hemicellulose degradation but do not produce H_2_. The inability of the PLS-DA model to identify *Fibrobacteraceae* as a key variable in the VIP, alongside the overrepresentation of *Ruminococcaceae*, suggests that factors beyond dietary composition are influencing the microbial community structure in the rumen. Given the shared role of *Fibrobacter* and *Ruminococcaceae* in cellulose degradation, a concurrent increase in the members from both taxa would be expected if diet were the primary determinant. However, the absence of this association in our findings indicates that other environmental or host physiological factors may play a more critical role in shaping the relative abundance and dominance of *Ruminococcaceae* within the rumen microbiome.

As discussed earlier, CH_4_ production in the rumen is primarily driven by archaea, particularly members of the *Methanobacteriaceae* family. In our study, *Methanobrevibacter* was the only genus that significantly differed between high and low CH_4_-emitting groups, with its abundance being higher in the low-emitting group. This limited detection of archaeal taxa may be attributed to methodological and technical constraints, as we employed primers targeting the V4 region of the 16S rRNA gene, which are designed to capture both bacterial and archaeal populations. However, the use of archaeal-specific primers targeting alternative regions of the 16S rRNA gene could provide a more comprehensive and accurate characterization of the archaeal community within the rumen (Pausan et al., 2019). Alternatively, researchers may utilize archaea-specific primers targeting the mcrA gene, which encodes the α-subunit of methyl-coenzyme M reductase, a key enzyme in methanogenesis. This approach enables a more precise assessment of archaeal communities, particularly in CH_4_-producing environments (Friedrich, 2005). Amplifying and sequencing the mcrA gene would not only enhance taxonomic resolution but also provide direct insights into the methanogenic potential and community composition in ruminants (Casañas et al., 2015). This approach is especially relevant in rumen studies, where a detailed understanding of methanogen diversity could contribute to strategies for mitigating CH_4_ emissions. While the 515F/806R primers used in this study effectively served the purpose of our broader study focused on large-scale microbial community comparisons and recovering global ecological patterns (Caporaso et al., 2011), we recognize that for studies specifically targeting the archaeal component of the rumen microbiome, and especially those investigating CH_4_ production, adopting the above-mentioned suggested approaches of utilizing archaea-specific primers would be highly beneficial. Another possible explanation for identifying only one archaeal ASV as discriminant is that the relative abundance of methanogens may not be a strong enough factor to differentiate high and low CH_4_ emitters. Research has indicated that gene expression levels, rather than the relative abundance of archaea, could serve as a more accurate marker for distinguishing between CH_4_ emission profiles (Roehe et al., 2016; Shi et al., 2014). This implies that functional activity of methanogens, rather than their population size, could play a more significant role in determining CH_4_ emission (Shi et al., 2014). reported a higher relative abundance of *Methanosphaera* spp. and a lower relative abundance of organisms belonging to the *Methanobrevibacter gottschalkii* clade in the low CH_4_ yield sheep. Even though these authors found some other shifts in subpopulations of methanogens, they concluded that the higher CH_4_ yield of the high CH_4_ emitting sheep was unlikely to be due to an increased relative abundance of these methanogens. They did find strong correlations between gene expression of methanogens and CH_4_ yield. It is therefore recommended for follow-up research to focus on gene expression rather than relative abundance to discuss the role of archaea in enteric CH_4_ production.

Our study elucidates the intricate interactions between dietary factors, rumen microbiota composition, and CH_4_ emission in dairy cattle, emphasizing the necessity for a more refined approach that extends beyond basic phenotype comparisons. The inherent variability across farm conditions complicates the identification of consistent microbial signatures associated with CH_4_ production. When designing studies in real-world farm settings, it is essential to account for the diverse management practices and environmental factors that influence microbial dynamics. Methodologies such as living labs offer a more accurate representation of microbial community responses to fluctuating on-farm conditions, thereby enhancing the precision of CH_4_ mitigation strategies. However, the dynamic and multifactorial nature of commercial farming presents significant challenges in identifying reliable rumen based “microbial biomarkers” for CH_4_ emission. Despite these obstacles, our findings underscore the critical need to incorporate on-farm variability to deepen our understanding of microbial dynamics and their contribution to CH_4_ production, ultimately improving the development of targeted mitigation strategies. While direct methanogen data is valuable (Patra et al., 2017; Danielsson et al., 2017), our findings underscore the need for a broader ecological perspective in understanding CH_4_ emissions in real-world settings. We show that shifts in the wider microbial community, including non-methanogenic taxa, are significantly associated with CH_4_ intensity produced by dairy cattle (Danielsson et al., 2017; Wallace et al., 2015; Bekele et al., 2010) likely reflecting environmental conditions and complex microbial interactions that indirectly regulate methanogenesis.

The pronounced susceptibility of CH_4_-associated rumen microbiota to environmental conditions necessitates a fundamental shift towards context-aware CH_4_ mitigation strategies in dairy farming. Interventions should leverage holistic approaches like optimized soil and grassland management, which impact the entire microbial community (Guo et al., 2024; Egan et al., 2018; Su et al., 2023; Chernov and Semenov, 2021). To advance sustainable solutions, future research must prioritize on-farm investigations into the effects of specific farm factors on key microbial groups involved in methanogenesis, including hydrogen producers, and their complex interactions. This integrative, field-based approach is crucial for achieving meaningful and consistent reductions in CH_4_ emissions.

## Data Availability

The datasets presented in this study can be found in online repositories. The names of the repository/repositories and accession number(s) can be found in the article/**Supplementary Material**.
