# Supplementary material for: Farm conditions shape microbial communities and their association with methane intensity in dairy cattle: insights from the rumen microbiome at the community level
Source: Front Microbiomes. 2025 Apr 30;4:1540197. doi: 10.3389/frmbi.2025.1540197 (PMC12993641; doi:10.3389/frmbi.2025.1540197)
Supplement: Supplementary file 2 [file DataSheet1.docx]

Supplementary Material

## Supplementary Figures


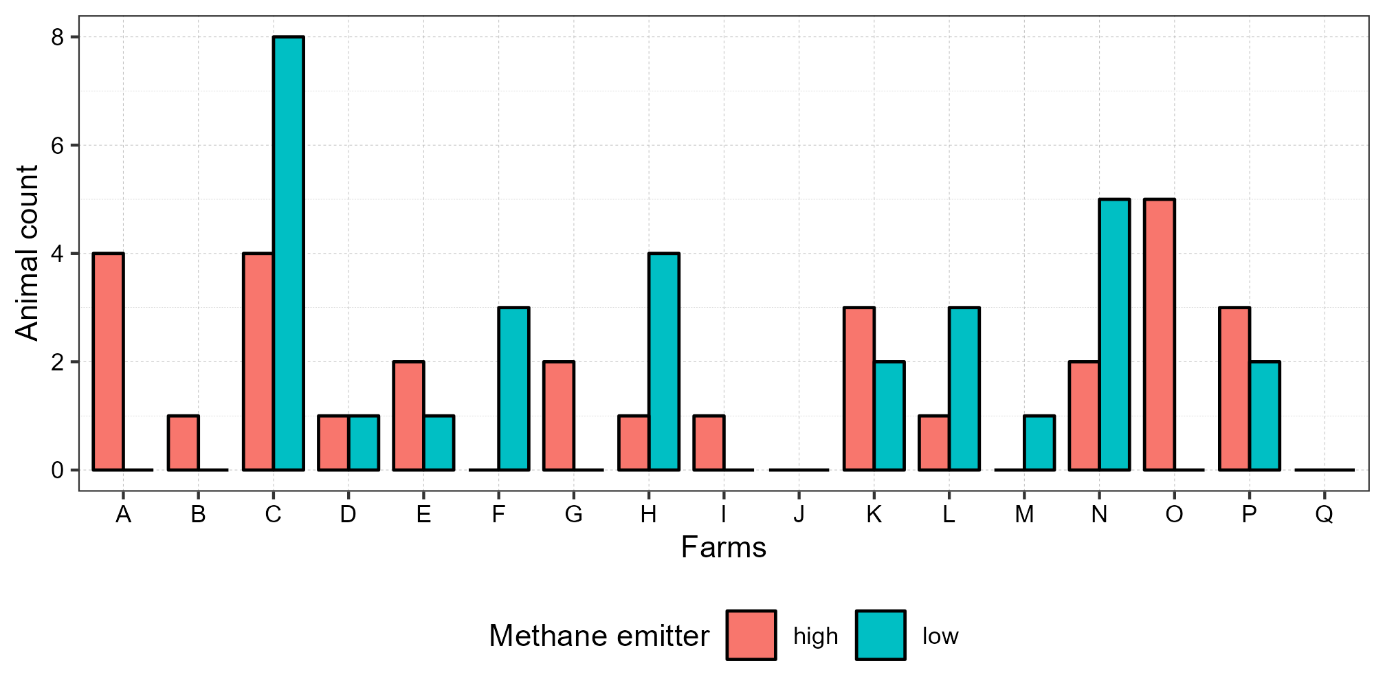


**Supplementary Figure 1.** The number of individual cows included in the balanced experimental design from each of the 17 farms.


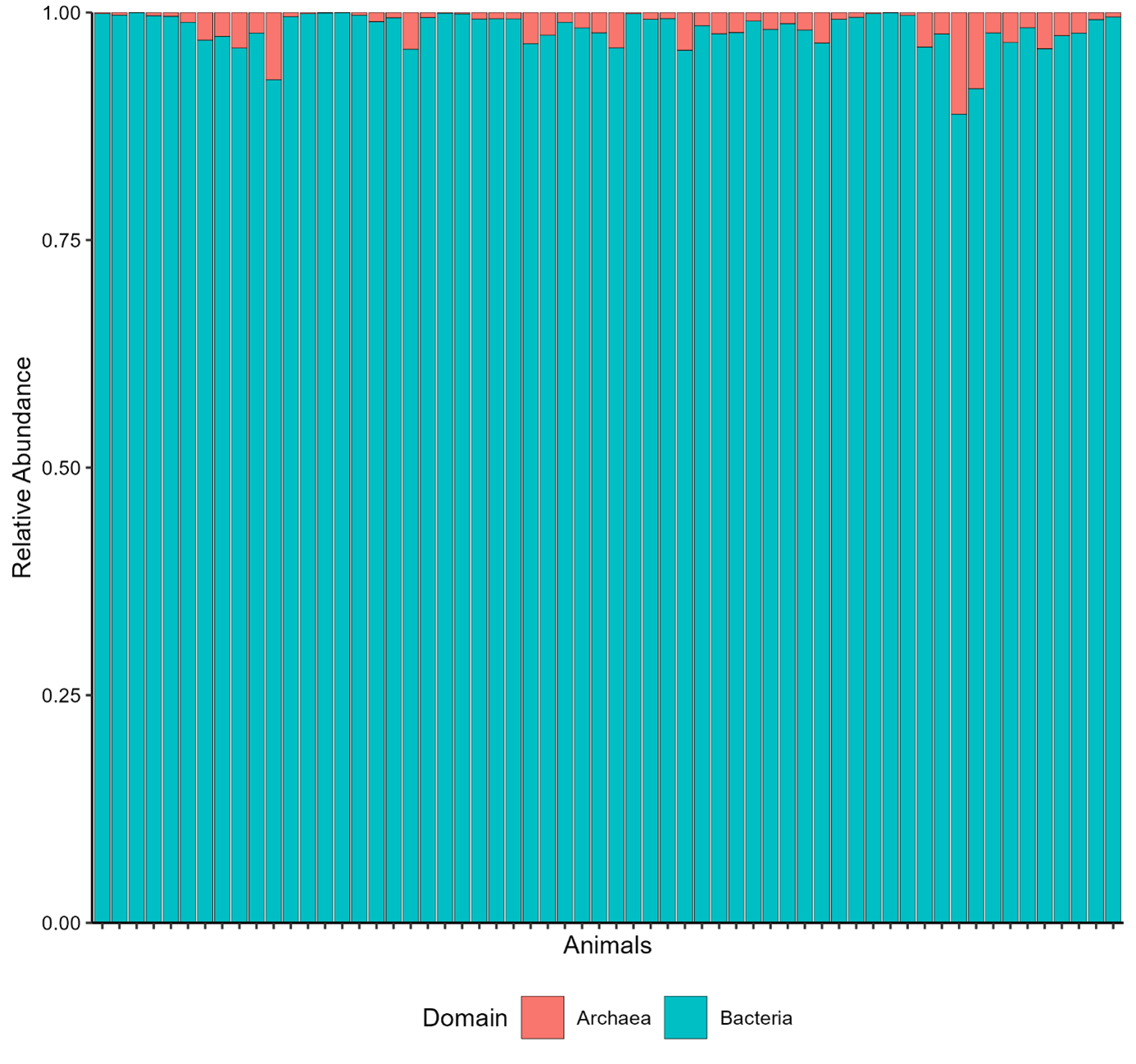


**Supplementary Figure 2.** Relative abundance of archaea and bacteria in the rumen fluid sample of 60 cows from the low and high emitters categories in the balanced experimental design before centered log-ratio normalization.


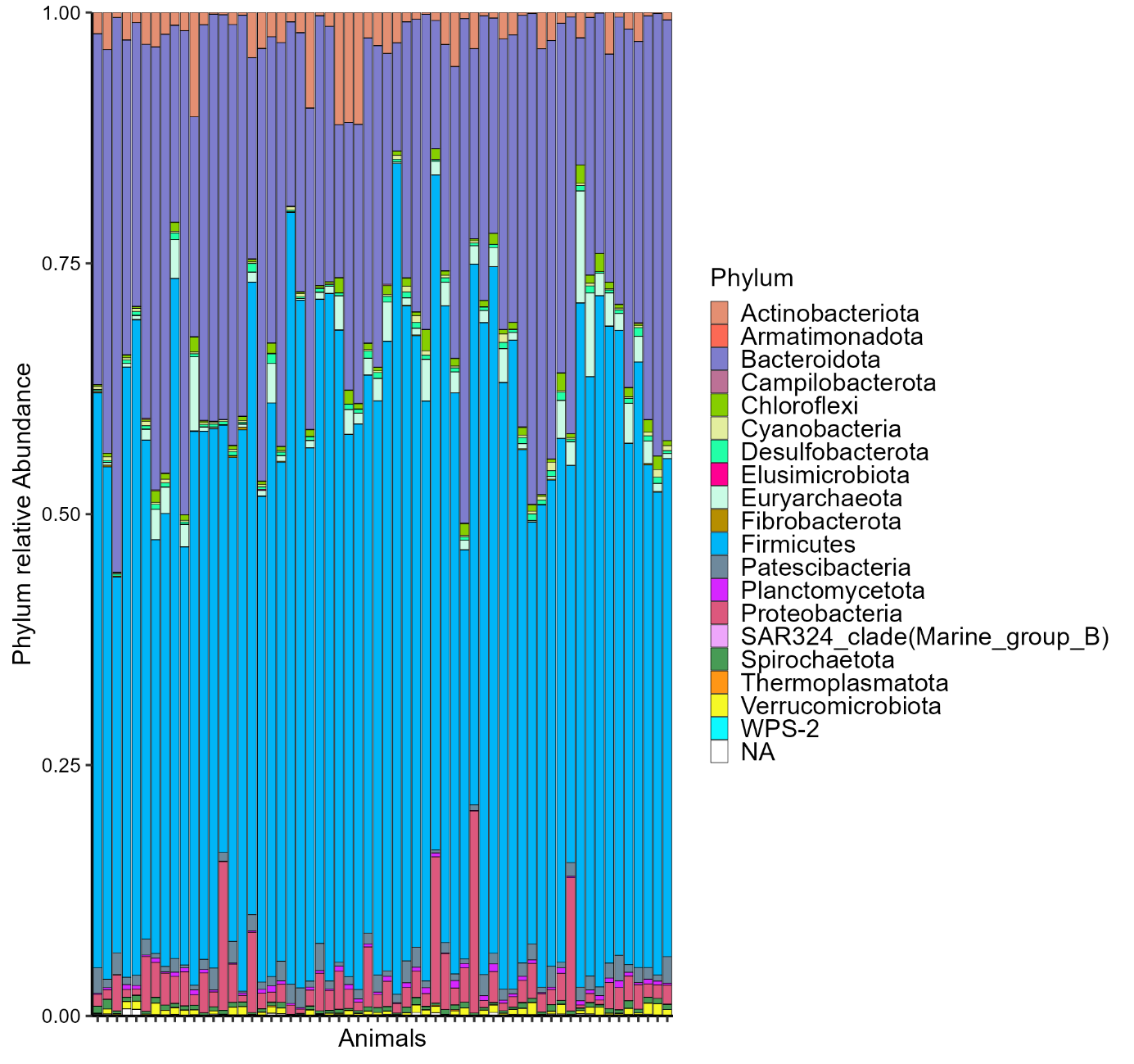


**Supplementary Figure 3.** Relative abundance of the rumen microbiota at the phylum level of 60 cows from the low and high emitters categories in the balanced experimental design before centered log-ratio normalization.


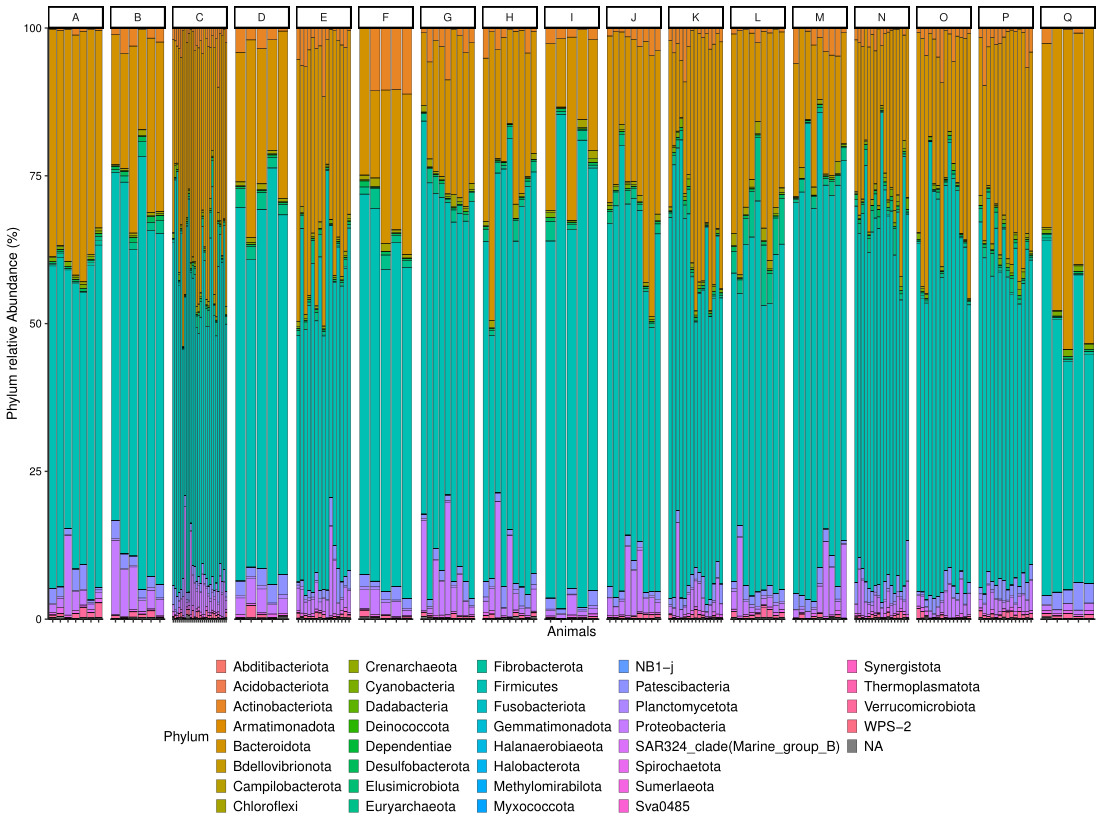


**Supplementary Figure 4.** Relative abundance of the rumen microbiota at the phylum level across the 17 farms (Farms A-Q).


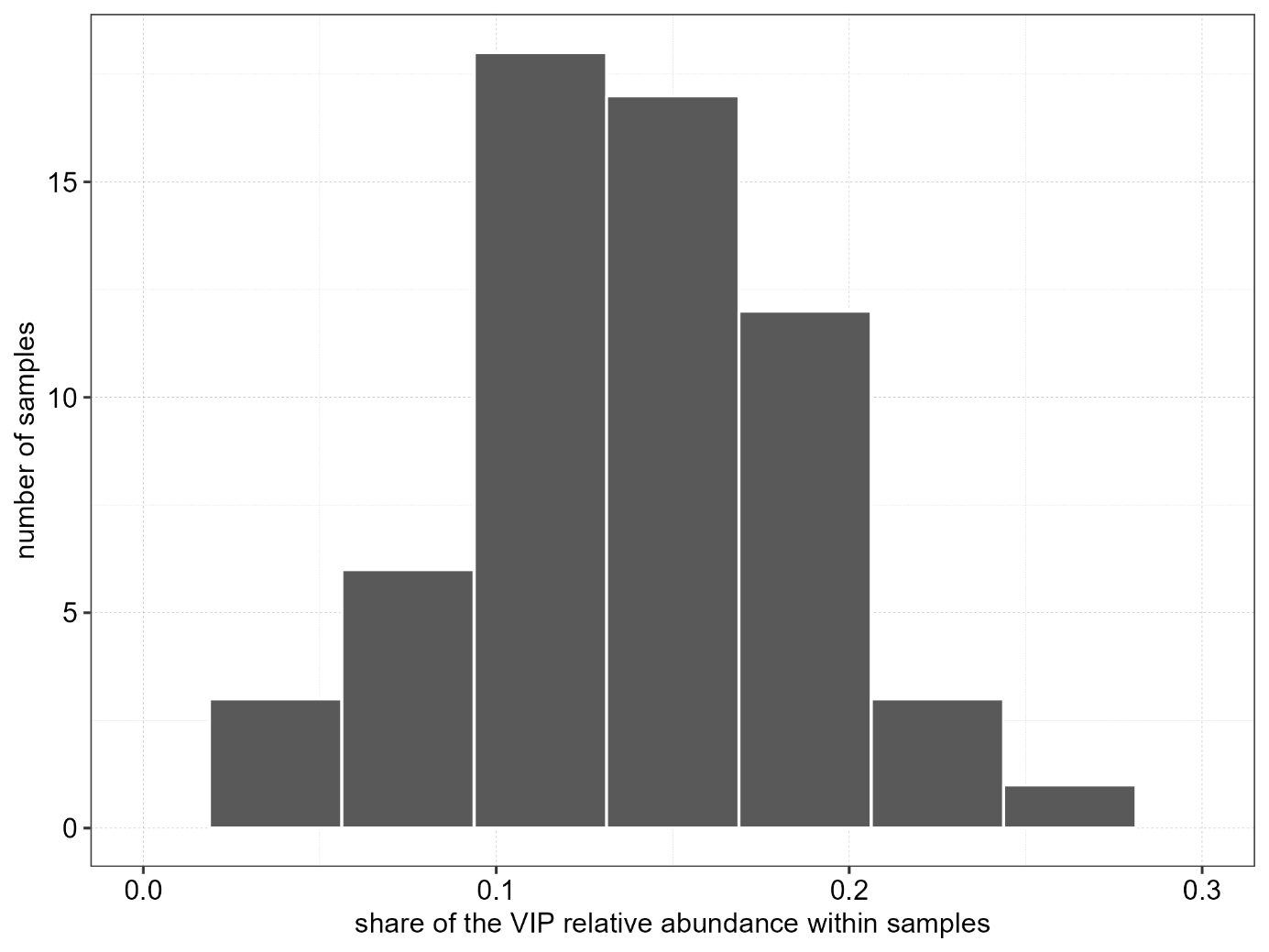


**Supplementary Figure 5.** Distribution of the share of the variable in projection relative abundance within samples for the 60 cows from the low and high emitters categories in the balanced experimental design.

## Supplementary Table

**Supplementary Table 1**: Overview of the farms visited, including the start and end date of each measurement period, date and time of rumen fluid sampling, average CH_4_ emission and milk production, lactation and parity information of the selected cows, and the diet composition on herd level.

*See Supplementary_Table_1.xlsx*
